# Effect of Storage Humidity on Physical Stability of Spray-Dried Naproxen Amorphous Solid Dispersions with Polyvinylpyrrolidone: Two Fluid Nozzle vs. Three Fluid Nozzle

**DOI:** 10.3390/pharmaceutics13071074

**Published:** 2021-07-13

**Authors:** Sonal V. Bhujbal, Yongchao Su, Vaibhav Pathak, Dmitry Y. Zemlyanov, Alex-Anthony Cavallaro, Eric J. Munson, Lynne S. Taylor, Qi (Tony) Zhou

**Affiliations:** 1 Department of Industrial and Physical Pharmacy, College of Pharmacy, Purdue University, 575 Stadium Mall Drive, West Lafayette, IN 47907, USA; sbhujbal@purdue.edu (S.V.B.); yongchao.su@merck.com (Y.S.); vpathak@purdue.edu (V.P.); munsone@purdue.edu (E.J.M.); lstaylor@purdue.edu (L.S.T.); 2 Pharmaceutical Sciences, Merck & Co., Inc., Rahway, NJ 07065, USA; 3 Birck Nanotechnology Center, Purdue University, 1205 West State Street, West Lafayette, IN 47907, USA; dzemlian@purdue.edu; 4 Future Industries Institute, University of South Australia, Adelaide, SA 5095, Australia; alex-anthony.cavallaro@unisa.edu.au

**Keywords:** amorphous solid dispersions, particle formulation, spray drying, physical stability, three fluid nozzle

## Abstract

In a spray drying operation, a two-fluid nozzle (2FN) with a single channel is commonly used for atomizing the feed solution. However, the less commonly used three-fluid nozzle (3FN) has two separate channels, which allow spray drying of materials in two incompatible solution systems. Although amorphous solid dispersions (ASDs) prepared using a 3FN have been reported to deliver comparable drug dissolution performance relative to those prepared using a 2FN, few studies have systematically examined the effect of 3FN on the physical stability. Therefore, the goal of this work is to systematically study the physical stability of ASDs that are spray-dried using a 3FN compared to those prepared using the traditional 2FN. For the 2FN, a single solution of naproxen and polyvinylpyrrolidone (PVP) was prepared in a mixture of acetone and water at a 1:1 volume ratio because 2FN allows for only one solution inlet. For the 3FN, naproxen and PVP were dissolved individually in acetone and water, respectively, because 3FN allows simultaneous entry of two solutions. Upon storage of the formulated ASDs at different humidity levels (25%, 55% and 75% RH), naproxen crystallized more quickly from the 3FN ASDs as compared with the 2FN ASDs. 3FN ASDs crystallized after 5 days of storage at all conditions, whereas 2FN ASDs did not crystallize even at 55% RH for two months. This relatively higher crystallization tendency of 3FN ASDs was attributed to the inhomogeneity of drug and polymers as identified by the solid-state Nuclear Magnetic Resonance findings, specifically due to poor mixing of water- and acetone-based solutions at the 3FN nozzle. When only acetone was used as a solvent to prepare drug-polymer solutions for 3FN, the formulated ASD was found to be stable for >3 months of storage (at 75% RH), which suggests that instability of the 3FN ASD was due to the insufficient mixing of water and acetone solutions. This study provides insights into the effects of solvent and nozzle choices on the physical stability of spray-dried ASDs.

## 1. Introduction

Dissolution and absorption are essential for oral dosage forms to be effective, but a majority of new drug candidates (>90%) belong to Biopharmaceutics Classification System (BCS) class II or IV with poor water solubility [1]. To improve solubility or dissolution of such drugs, several formulation techniques have been developed, such as amorphous solid dispersions, microemulsions, co-crystals, nanoparticles, salt formation, and cyclodextrin complexation [2,3]. In the past two decades, amorphous solid dispersions (ASD) have gained popularity for their ability to improve drug dissolution and for their bioavailability, especially for BCS class II drugs [4].

An ASD is a molecular dispersion of a drug in an excipient (in most cases, a polymer) [5]. In the amorphous form, a drug usually possesses a higher apparent solubility and dissolution rate than in its crystalline form. The lower solubility of the crystalline form is due to the additional energy required to remove drug molecules from a crystal lattice during solubilization [6,7]. Since a majority of small-molecule drugs are physically unstable in amorphous form, an ASD is used to prevent crystallization. ASDs can be prepared using different techniques such as hot-melt extrusion, quench cooling from the melt, spray drying, electrospinning, and solvent evaporation [8]. Spray drying has increasingly been applied for manufacturing ASDs over the last decade, particularly for thermo-sensitive drugs that cannot withstand high-temperature methods such as hot-melt extrusion [9]. The technique involves atomizing a feed solution of drug and polymer followed by its rapid drying to particles in the amorphous form [9]. Spray drying is a continuous process with scale-up capability [10]. It is currently employed in the manufacture of several commercial ASDs [11].

**Figure 1:**
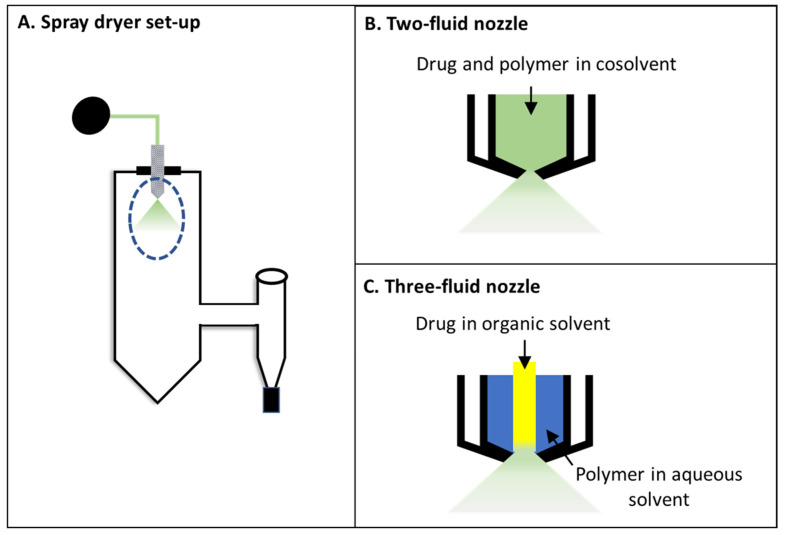
Schematic of a typical (**A**) spray dryer setting; (**B**) two-fluid nozzle; (**C**) three-fluid nozzle (with internal mixing).

There are several equipment variables associated with the spray dryer that affect ASD product properties [8]. The spray nozzle (Figure 1A), which is used for atomizing the feed solution, is one such critical variable. In the commonly used two-fluid nozzle (2FN), the feed solution is pumped through an inner channel and the atomizing gas passes through a concentric outer channel (Figure 1B) [12,13]. Therefore, only a single feed solution (necessarily made of compatible components and solvents) is allowed in the 2FN configuration. On the other hand, a three-fluid nozzle (3FN) allows two feed solutions to be separately pumped through two passages (Figure 1C). Such a configuration allows spray drying of two different materials in two incompatible solution systems [14]. For example, in the work of Sunderland et al., omeprazole sodium and Eudragit L100 (EL100) could not have been spray-dried using a 2FN. The two components could not form a co-dissolved feed solution, showing drug precipitation and discoloration due to omeprazole’s instability at low pH (EL100 solution has a pH of 2.8). However, when the two components were spray-dried using a 3FN, the individual solutions of drug and polymer remained stable during the process [14].

For ASDs, the two most critical properties are the extent of drug release upon dissolution and drug stability upon storage (e.g., crystallization). ASDs prepared using a 3FN have been reported to deliver comparable drug dissolution performance relative to those prepared using a 2FN [15]. As shown by Chen et al., an ASD of tolbutamide with hydroxypropyl methylcellulose prepared using a 3FN showed higher drug release and faster dissolution rate than the raw crystalline drug [15]. However, few studies have systematically examined the effect of a 3FN spray drying on the physical stability of ASDs upon storage. The two separate channels, each of the drug and polymer solution combined with the relatively shorter residence time at the nozzle tip where they mix, can result in ASDs with a heterogeneous drug-polymer distribution. Such heterogeneity in ASDs is known to facilitate phase separation, subsequently leading to crystallization and negating the ASD solubility advantage [16,17]. The goal of this work is to address this knowledge gap by determining the storage stability of ASDs that are manufactured by spray-drying using a 3FN compared to those prepared using the traditional 2FN. In case of a significant discrepancy in the physical stability of the two samples, the study also aimed to explore fundamental understanding of the cause of physical instability. Naproxen and polyvinylpyrrolidone (PVP) were selected as a model drug-polymer system for this study.

## 2. Experimental Section

### 2.1. Materials

Naproxen was purchased from Attix Pharmaceuticals (Toronto, Ontario, Canada). PVP was supplied by BASF Corporation (Florham Park, NJ, USA). Acetone (HPLC grade) was purchased from Sigma–Aldrich (St. Louis, MO, USA).

### 2.2. Formation of Solid Dispersion via Spray Drying

Spray-dried ASDs were prepared using a Büchi 290 spray dryer (Büchi Labortechnik AG, Flawil, Switzerland) equipped with either a 2FN or a 3FN. A 2FN has only one fluid inlet along with a gas inlet. Since the 2FN allows only one solution inlet, a solution of naproxen and PVP in 1:4 ratio was prepared in an acetone:water (1:1 volume ratio) co-solvent with a total solid concentration of 50 mg/mL. In contrast, the 3FN allows simultaneous entry of two separate fluid lines. Naproxen and PVP solutions were prepared individually in acetone (4 mg/mL) and water (16 mg/mL), respectively. The rest of the spray drying operating parameters were same for both ASDs samples and were set to the following values: feed rate 4 mL/min, atomizer setting 700 L/h, inlet temperature 150 ± 2 °C, aspirator 35 m^3^/h, outlet temperature 80 ± 2 °C. The spray-dried ASD was immediately collected and transferred into 20 mL glass vials. These vials were placed in desiccators until analysis.

### 2.3. Storage Stability Study

The open glass vials containing samples were stored in desiccators. The desiccators were maintained at three different RH conditions of 20%, 55% and 75%, and were kept at room temperature (22–25 °C).

### 2.4. Powder X-ray Diffraction

Powder samples were analyzed using a Rigaku SmartlabTM diffractometer (Rigaku Americas, TX, USA) to obtain X-ray diffraction patterns. The instrument used a Cu-Kα radiation source operating at 40 kV voltage and 44 mA current and a D/tex ultra-detector. The samples were evenly spread over a designated area on a glass slide. The glass slide was placed on the sample holder of the X-ray diffractometer for analysis. Each sample was scanned over the 2θ range of 4–40° at a rate of 4°/min, and the resulting diffractograms had a resolution of 0.02°.

### 2.5. Thermogravimetry

Using a PerkinElmer TGA 4000 instrument (Waltham, MA, USA), each sample (~5 mg) was heated under a nitrogen gas environment from 35 °C to 170 °C at 10 °C/min and its weight was continuously recorded. The sample volatile content was calculated as the percent weight loss between 35 °C and 170 °C [18].

### 2.6. Dynamic Vapor Sorption (DVS)

Moisture absorption at different relative humidity (RH) levels was measured for ASDs and pure components using a symmetrical gravimetric analyzer (SGA-100; VTI Corporation, Hialeah, FL, USA). Each sample was dried prior to measurement by heating at 40 °C under dry nitrogen purging. The drying step was programmed to conclude if less than 0.01% change in sample weight was observed over 2 min or if the total drying time reached 180 min. After this, the sample was equilibrated in steps at increasing values of RH at 25 °C. Starting at 5% RH, the humidity was increased up to 95% RH in 10% intervals. The equilibration criteria was less than 0.01% change in sample weight over 5 min at a maximum of 180 min for each step. Subsequently, the sample was equilibrated at decreasing RH values in steps of 10% from 95% to 5% to obtain the desorption profile.

### 2.7. Scanning Electron Microscopy (SEM)

A NOVA nanoSEM (FEI Company, Hillsboro, OR, USA), a field emission scanning electron microscope equipped with a high-resolution through-the-lens detector (TLD), was used to obtain images of microscopic particle morphologies of ASD samples. To prepare samples for analysis, a thin layer of the powder was attached to a sample holder using two-sided carbon tape and coated using a 208 HR Cressington Sputter Coater (Watford, UK) with platinum for 60 s. The sample images were captured at an accelerating voltage of 5 kV. The sample was focused at a working distance of ~5 mm.

### 2.8. Polarized Light Microscopy

Polarized light microscopy was used to qualitatively characterize the crystallinity of ASD samples based on the observation of birefringence. Each powder sample was evenly applied on a glass slide and observed through a Nikon Eclipse E600 POL cross-polarized light microscope (10× objective) (Melville, NY, USA). The sample image was captured by a Nikon DS-Ri2 camera.

### 2.9. Time-of-Flight Secondary Ion Mass Spectrometry (ToF-SIMS)

ToF-SIMS (nanoToF instrument, Physical Electronics Inc., Chanhassen, MN, USA) was used to analyze the surface composition of ASD powders [19]. The instrument is equipped with a pulsed liquid metal ^79+^Au primary ion gun and is capable of dual charge neutralization via an electron flood gun and 10 eV Ar^+^ ions. The analysis was performed at 30 kV energy and in positive SIMS mode. The instrument was optimized for spatial resolution using an “unbunched” Au1 setting. For each sample, data were collected using five random replicates where each involved a 100 × 100 μm^2^ area and a 2-min acquisition time.

Surface composition overlays were obtained from WincadenceN software (Physical Electronics Inc., Chanhassen, MN, USA). On the raw image, the region-of-interest analyses were performed. In order to extract the particle surface chemistry of a sample, the mass spectra data were collected only from within the region of interest. For calibration and peak selection, characteristic peak fragments were chosen for naproxen (C_13_H_13_O^+^) and PVP (C_6_H_10_NO^+^). The integrated peak values of these ion fragments were normalized to the total secondary ion intensity and used to perform a semi-quantitative comparison of particle surface chemistry.

### 2.10. X-ray Photoelectron Spectroscopy (XPS)

The surface composition of ASD solids was evaluated quantitatively using X-ray photoelectron spectroscopy (XPS) (AXIS Ultra DLD spectrometer, Kratos Analytical Inc., Manchester, UK) [19,20,21]. The detailed XPS method has been described previously [19]. XPS data analysis was conducted using CasaXPS software (version 2313 Dev64). Curve-fitting was performed following a Shirley background subtraction using model peaks obtained from pure compounds. The atomic concentrations of the elements in the near-surface region were estimated by the CasaXPS software by factoring in the Scofield atomic sensitivity factors and inelastic mean free path of photoelectrons (~10 nm).

### 2.11. Solid-State NMR Spectroscopy

Solid-state NMR (ssNMR) was utilized to probe the homogeneity of naproxen and PVP in the ASD [22] after the differential scanning calorimetry results for the same were inconclusive, likely due to the lower drug loading (Figure S1).

All experiments were carried out on a Bruker 400 MHz Avance III HD spectrometer (Billerica, MA, USA), which is situated at Biopharmaceutical NMR Laboratory (BNL), Pharmaceutical Sciences, Merck & Co. (West Point, PA, USA). ^1^H longitudinal (or spin-lattice) relaxation time in the laboratory and rotating frame, T_1_ and T_1rho_, respectively, is measured. ^13^C-detected inverse recovery and spin lock sequences are utilized to measure T_1_ and T_1rho_, respectively [23]. Primary experimental parameters include a MAS frequency of 12 kHz, sample temperature at 294 K, an acquisition time of 17.2 ms, and a recycle delay of 2.5 s. A contact time of 2 ms, linear ramp, an ^1^H 90-degree pulse at 2.5 µs, and 50 kHz Hartmann–Hahn matching condition was utilized for ^1^H-^13^C cross-polarization (CP). An ^1^H SPINAL decoupling at 83 kHz was employed during acquisition, and a spinlock field of 100 kHz was applied for T_1rho_ measurement. As-received ASD powder samples were packed in 4 mm rotors for all experiments. All ^13^C spectra were referenced to adamantane and processed in TopSpin.

### 2.12. Statistical Analysis

An independent t-test was used to determine the statistical difference between two groups. The statistical analysis was conducted using SPSS™ software (SPSS Inc. IBM Corporation, New York, NY, USA). The data are presented as mean ± standard deviation.

## 3. Results

### 3.1. Physical Characterization of ASDs upon Spray-Drying

**Figure 2:**
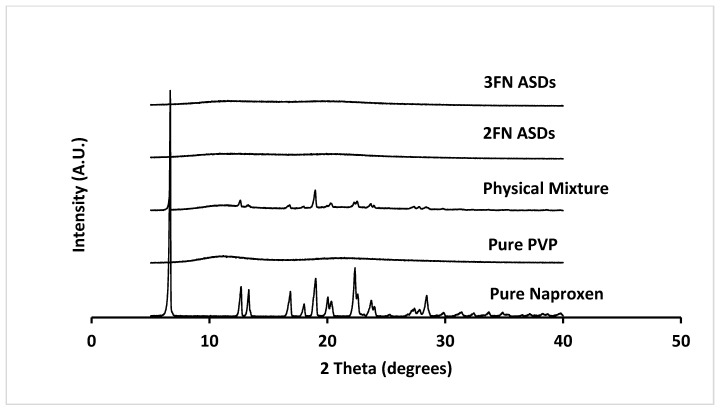
X-ray diffraction patterns of as-received naproxen, neat PVP, naproxen-PVP physical mixture and two naproxen-PVP dispersions (2FN and 3FN) right after spray drying.

PXRD was used to test the conversion of crystalline naproxen into its amorphous form, to confirm the successful formation of ASDs. The as-received naproxen showed sharp PXRD peaks indicating its crystalline nature (Figure 2). In contrast, 2FN and 3FN solid dispersions of naproxen-PVP did not exhibit any crystalline peaks, which demonstrated that both were X-ray amorphous immediately following production.

**Figure 3:**
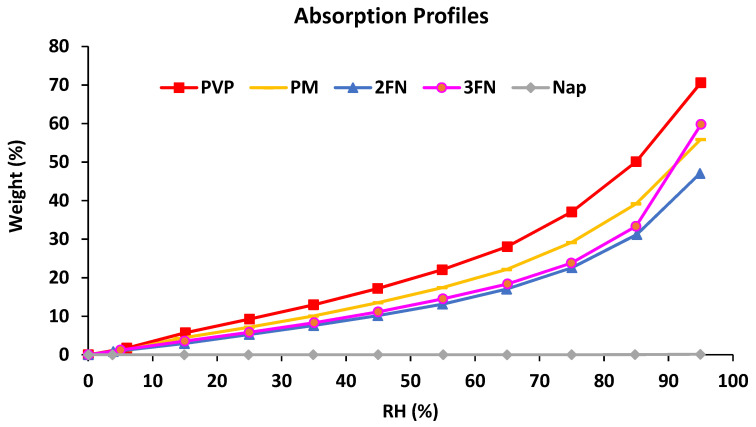
Moisture absorption profiles of raw materials, physical mixture, and two naproxen-PVP dispersions (2FN and 3FN) at 25 °C.

DVS profiles (Figure 3) of the 3FN and 2FN ASD samples indicated a difference in moisture absorption only at the highest RH point of around 95% RH. The 3FN sample crystallized at the end of the experiment. Interestingly, the physical mixture containing crystalline naproxen shows higher moisture sorption than the 2FN ASD. This is likely due to intermolecular interactions between naproxen and PVP in the 2FN ASD that can reduce water sorption. Crowley et al. obtained similar results wherein a reduction in water sorption was observed due to increased drug-polymer interaction for PVP ASDs [24]. At this point, the higher water uptake of the 3FN sample at 95% RH is consistent with physical instability.

**Figure 4:**
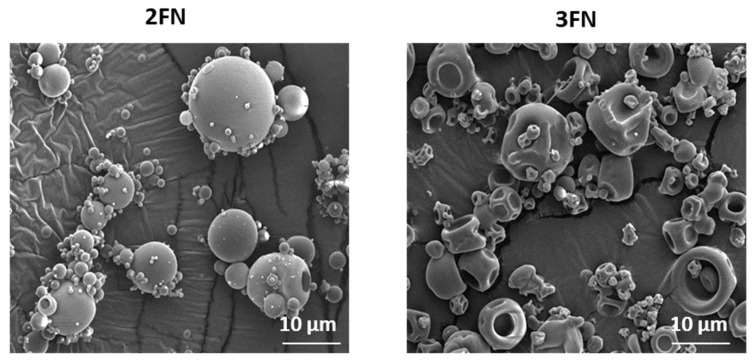
Representative scanning electron microscopy images of naproxen-PVP dispersions (2FN and 3FN) immediately after spray drying.

TGA data indicated a slightly higher, but not significant (*p* > 0.05), volatile content for the 3FN ASDs (7.04 ± 0.06% *w*/*w*) as compared to the 2FN (5.71 ± 0.30% *w*/*w*). The two batches had similar size ranges, but very different particle shapes and surface textures, according to SEM images. The 2FN ASD particles were spherical and smooth, while those of the 3FN were dimpled (Figure 4).

### 3.2. Physical Stability of ASDs upon Storage

**Figure 5:**
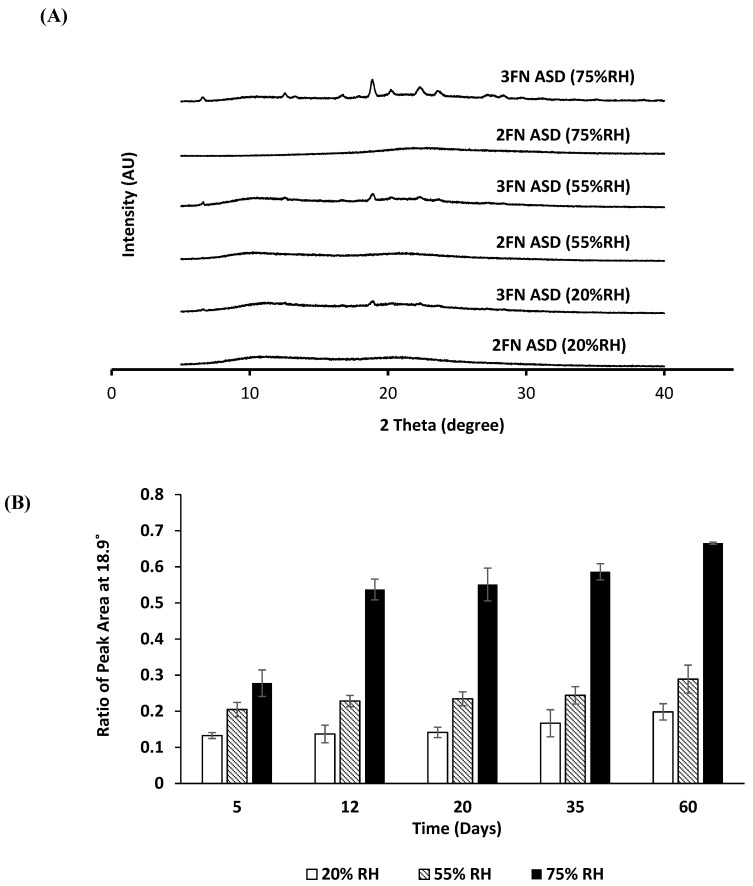
(**A**) X-ray diffraction patterns of naproxen-PVP dispersions (2FN and 3FN) after 60 days storage at 20, 55, and 75% RH; (**B**) the ratio of area under the PXRD pattern for the 3FN naproxen-PVP dispersion and pure crystalline naproxen at 2θ = 18.9° (mean ± SD, *n* = 3).

The PXRD patterns of the 2FN and 3FN ASD samples stored at 20, 55, and 75% RH for 2 months are shown in Figure 5A. The 2FN samples did not show XRD peaks at any RH storage condition; while the 3FN samples showed signs of crystallization at all RH conditions. The intensity of the peaks for the 3FN samples increased with an increase in the storage RH. The most prominent peak was observed near 19° for all 3FN samples, which corresponds to a characteristic XRD peak of the naproxen crystal. To obtain a semi-quantitative estimate of the level of crystallinity, the area under the curve of this characteristic peak was measured. The ratio of this intensity to the corresponding peak intensity of the naproxen-PVP physical mixture was calculated and plotted (Figure 5B). The relative peak intensity ratio provides an estimate of the extent of crystallization in the dispersion. As shown in Figure 5B, an increase in crystallinity was observed over time at all three RH conditions for the 3FN samples.

**Figure 6:**
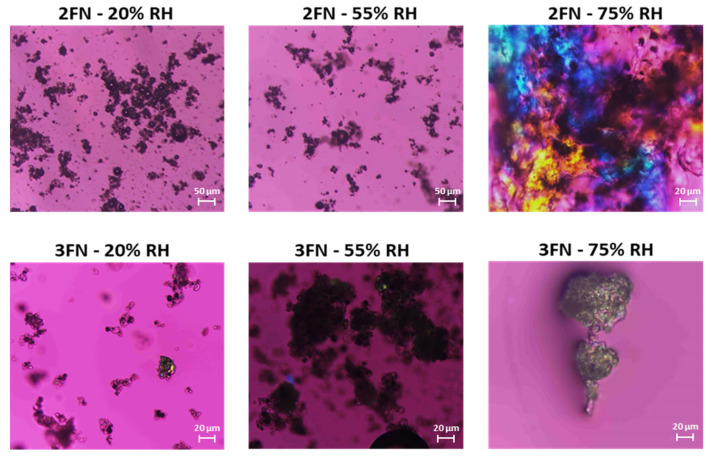
Polarized light microscopy images of naproxen-PVP dispersions (2FN and 3FN) stored under different humidity levels after 60 days.

Figure 6 shows the representative polarized light microscopy images. The 20% and 55% RH samples of 2FN ASDs showed no birefringence, which confirmed the absence of crystallinity. All the 3FN ASD samples showed birefringence, confirming the crystallization of naproxen in the dispersions when stored under the studied RHs. However, notable birefringence was observed in the 2FN ASD sample stored at 75% RH despite the PXRD pattern showing no crystallinity peaks. This is likely because of the presence of a small amount and/or a small size of crystals formed due to local phase separation. To visualize these potential small crystals, the samples were further studied using scanning electron microscopy.

**Figure 7:**
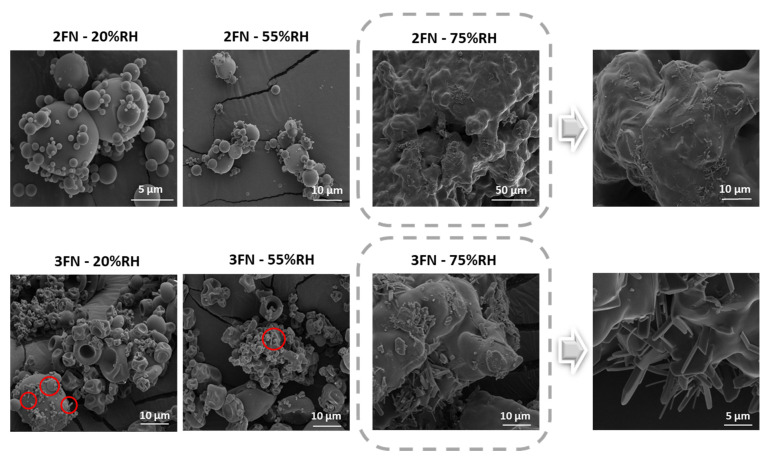
SEM images of naproxen-PVP dispersions (2FN and 3FN) stored under different RH after 60 days. Red circles indicate crystals.

The SEM images of different ASDs are shown in Figure 7. The 20% and 55% RH samples of the 2FN ASDs showed no crystals after two months; however, the 75% RH sample images exhibited some small crystals on the particle surface. SEM images confirmed the crystallinity of 2FN samples stored under 75% RH observed by polarized light microscopy. PXRD did not detect the crystallinity, most likely due to its lower sensitivity; PXRD typically has a detection limit for crystalline drugs of around 5% [25]. Needle-shaped crystals were observed on the particle surface for 3FN ASDs stored at all RH conditions (Figure 7).

### 3.3. Drug-Polymer Surface Distribution

**Figure 8:**
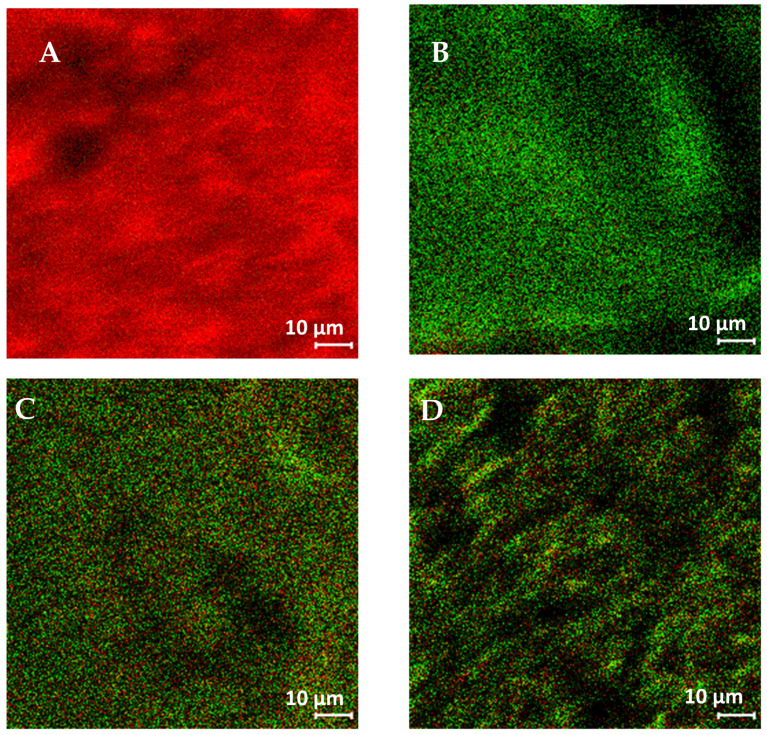
Distribution of naproxen (red) and PVP (green) measured by ToF-SIMS on the particle surface of (**A**) crystalline naproxen; (**B**) neat PVP; (**C**) 2FN ASD and (**D**) 3FN ASD (scale bar represents 10 µm).

The surface composition of ASDs was analyzed and compared using ToF-SIMS (Figure 8) and XPS (Table 1). Based on visual observation of the ToF-SIMS images, both dispersions indicated similar surface compositions. To confirm this observation, the surface composition was further evaluated by XPS.

The XPS data demonstrated that the composition was essentially homogeneous on the particle surface for both 2FN and 3FN ASDs, and that the measured surface concentrations of naproxen and PVP (as determined by curve-fits of the C 1s) were comparable to the theoretical concentrations (calculated by normalizing the relative carbon atom proportion in the formulation). Statistical analysis indicated no difference between the surface composition of the 2FN and 3FN ASD formulations (*p* > 0.05). The molecular miscibility between drug and polymer in these ASDs at a sub-100 nm resolution was characterized by ssNMR studies.

### 3.4. Homogeneity of Drug and Polymer Determined as by Solid-State NMR (ssNMR)

In recent years, ssNMR has been utilized to assess the homogeneity of drug substances and polymer in ASDs at a high-resolution and to successfully correlate such microscopic attributes to the formulation processes [23,26,27,28,29]. ^1^H T_1_ and T_1rho_ values of individual components were measured as described in the method section and were compared to evaluate the homogeneity. When drug and polymer exhibit highly similar T_1_ values, they are regarded as well-mixed at the length scale of approximately 20–100 nm. Due to the different time (or frequency) scale, an identical T_1rho_ value of the two components represents a good mixing at approximately 1–20 nm. Distinct relaxation times suggest a lack of homogeneity at the corresponding domain sizes. The domain sizes can be derived using a generic spin diffusion equation [23,29]. As shown in Table 2, 2FN exhibits highly identical T_1_ (and T_1rho_) of naproxen and PVP, suggesting a high degree of homogeneity within the ASD at a length scale of less than 9 nm. Interestingly, 3FN shows a similar ^1^H T_1_ of the two components but distinct ^1^H T_1rho_ values, suggesting a partially homogeneous system. It is worth mentioning that 2FN and 3FN have identical one-dimensional ^13^C spectra (data not shown), suggesting that the two ASDs do not have distinctly different molecular structures and interactions, regardless of the level of mixing [28,30].

## 4. Discussion

The goal of the study was to evaluate the physical stability of ASDs prepared by the 3FN and 2FN with the spray drying processes. Although the physical characterization of the two batches immediately after spray drying did not indicate any significant difference in drug amorphization (as analyzed by PXRD), a significant difference was found in the 2-month storage stability. The 3FN ASD exhibited crystallization under all tested humidity conditions from early time points (5th day in storage), whereas the 2FN ASD did not exhibit any crystallization at 55% RH up to two months. As both ASDs were amorphous upon preparation, and were stored and analyzed under same conditions, the cause of the poorer stability of 3FN ASD is likely the presence of a heterogeneous drug-polymer distribution in the ASDs. Inhomogeneity in drug-polymer distribution is known to result in an increased tendency of the drug to crystallize [31,32]. In order to understand the mechanism of physical instability of the 3FN ASD, the surface composition, hygroscopicity and drug-polymer homogeneity of the relevant samples were characterized.

Spray drying has been shown to produce particles with a heterogeneous distribution of drug and polymers. Vehring et al. discussed the correlation of surface enrichment of a component with its Peclet number [33]. Peclet number, given by the equation below, is directly proportional to the solvent evaporation rate (*k*) and inversely related to diffusivity of the component of interest (*D_i_*).

(1) $$P_{ei}=\frac{k}{8D_{i}}$$

Simulations by Vehring et al. suggested that a higher Peclet number of a component results in its higher surface enrichment. For example, when the drying kinetics are faster than the component diffusivity, the component tends to concentrate on the outer surface. Previous studies reported that the poorly water-soluble rifampicin [34] and azithromycin [35] were enriched on the particle surfaces when co-spray dried with the water-soluble colistin in a solvent of ethanol/water (1:1). For ASDs, if the drug is enriched on the surface of spray-dried particles, crystallization can be facilitated, particularly when the particles are exposed to moisture [16,36]. However, no significant difference was observed in the surface composition of the two ASD formulations and no drug enrichment was observed on the surface. Therefore, it is unlikely that surface drug enrichment is the cause of physical instability in the 3FN ASD.

Exposure of ASDs to moisture upon storage is also known to accelerate phase transformation [16]. A clear effect of moisture on drug crystallization can be seen in this study for the 3FN ASDs. At any time point, the extent of crystallinity was the highest for the 3FN ASD exposed to 75% RH, followed by 55% RH and 20% RH. Both the samples seemed to have a similar hygroscopicity as observed from their TGA and DVS profiles, although the 3FN ASD has slightly higher volatile contents. The 3FN ASD also exhibited a higher particle surface area despite a larger particle size (Table S1). Higher volatile content with higher surface area may result in poorer physical stability of ASDs during storage; we hypothesize that the heterogeneous drug-polymer distribution occurred in the 3FN ASDs during the manufacturing process was the primary contributor to the instability of the 3FN ASDs. With inhomogeneity, regions of the ASD are relatively drug-rich, and there is a reduction in the inhibitory effect of the polymer on drug crystallization. An inhomogeneous distribution of drug and polymer could explain why the 3FN ASDs undergo drug crystallization despite a high polymer load (i.e., 80% *w*/*w*). This higher extent of inhomogeneity of drug and polymer in the 3FN ASDs was confirmed by ssNMR relaxation measurement at a sub-10 nm domain size. In order to understand how the manufacturing process resulted in two batches with such distinct physical stability, a closer look was taken at the manufacturing variables.

**Figure 9:**
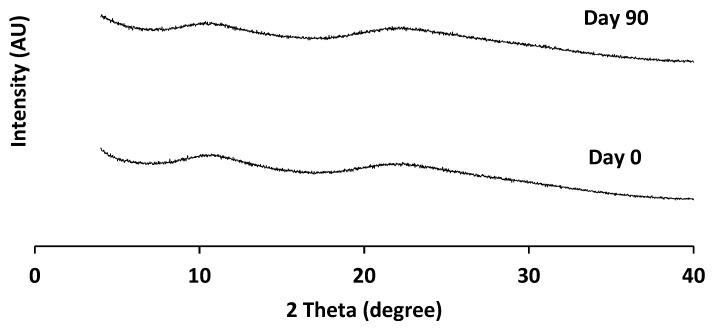
X-ray diffraction patterns of naproxen-PVP dispersions prepared by 3FN using acetone as a solvent for both drug and polymer, when measured immediately after spray drying (Day 0) and after 90 days of storage at 75% RH at 25 °C.

All the equipment and process variables for spray drying the two batches were the same. The only difference between the two batches was the nozzle used: a 3FN with two inlets for two separate lines of the drug and the polymer solutions, and an N with a single inlet for a single drug–polymer solution. Therefore, a possible reason for heterogeneous drug–polymer distribution in the 3FN ASD particles could be insufficient mixing of the drug (in organic solvent) and the polymer (in water) solutions due to a short mixing time prior to particle solidification during spray drying. For the 2FN system, the drug and polymer were dissolved in the same solution, lessening the chance of heterogeneity. In order to determine if the poor physical stability of the 3FN ASD was due to the insufficient mixing of the organic and aqueous solvents for the drug and the polymer, respectively, another batch of 3FN ASD was prepared using acetone as solvent for both the drug and the polymer. Each solution was fed into the system with separate lines as before, and all the processing parameters were identical to the other 3FN ASD. PXRD data of this batch demonstrated no crystallinity even after 90 days of storage in 75% RH (Figure 9). Thus, insufficient mixing of the drug and the polymer solutions prior to the formation of ASD particles appears to lead to heterogeneity and create ASDs with compromised stability. Such difference in mixing between 2FN and 3FN ASD particles could also be the cause of different surface morphology. Therefore, although the 3FN has the potential to prepare stable ASDs, the selection of appropriate solvents is a crucial factor to maintain a homogenous dispersion of drug and polymer in an ASD formulation and to achieve satisfactory physical stability during storage.

## 5. Conclusions

Amorphous solid dispersions of naproxen and PVP were prepared using 2FN and 3FN. Both ASDs were PXRD amorphous immediately after spray drying. Upon storage at different humidity conditions, naproxen crystallized more quickly in the 3FN ASDs than in the 2FN ASDs. This relatively higher crystallization tendency of the 3FN ASDs was attributed to the inhomogeneous distribution of drug and polymer due to insufficient mixing of the drug (in the organic solvent) and polymer (in water) feed solutions prior to drying. For the ASDs prepared using the 3FN, the choice of solvents significantly affected the extent of effective mixing. The 3FN ASDs prepared using the same solvent for the drug and polymer solutions resulted in a physically stable ASD that did not crystallize for more than 3 months at 75% RH. This study provides a fundamental understanding on how solvent and nozzle type affect the physical stability of spray-dried ASDs.

## Figures and Tables

**Table 1:** Surface composition of the naproxen-PVP dispersions (2FN and 3FN) as measured by XPS (mean ± SD, *n* = 5).

Formulation	Theoretical Surface Composition (% Weight)		Practical Surface Composition (% Weight)	
	Naproxen	PVP	Naproxen	PVP
2FN	20	80	20 ± 1	80 ± 1
3FN	20	80	17± 2	82 ± 2

**Table 2:** Molecular homogeneity between naproxen and PVP in the 2FN and 3FN ASDs from ssNMR measurements.

Formulation		T_1_ (s)	Homogeneous	Domain Size (nm)	T_1rho_ (ms)	Miscible	Domain Size (nm)
2FN	Naproxen	2.58 ± 0.09	Yes	112	17 ± 2	Yes	9
	PVP	2.64 ± 0.04			18 ± 3		
3FN	Naproxen	2.97 ± 0.43	Yes	120	18 ± 2	No	9
	PVP	3.05 ± 0.09			27 ± 1		11

## Data Availability

The data sets used and/or analyzed during the current study are available from the corresponding author on reasonable request.

## Supplementary Materials

The following are available online at https://www.mdpi.com/article/10.3390/pharmaceutics13071074/s1, Figure S1: A representative DSC plot of the immiscible (as confirmed by ssNMR) 3FN sample, Table S1: Particle sizes (*n* = 100) as measured from SEM images using the ImageJ software.
