# Supplementary material for: Effect of Storage Humidity on Physical Stability of Spray-Dried Naproxen Amorphous Solid Dispersions with Polyvinylpyrrolidone: Two Fluid Nozzle vs. Three Fluid Nozzle
Source: Pharmaceutics. 2021 Jul 13;13(7):1074. doi: 10.3390/pharmaceutics13071074 (PMC8309117; doi:10.3390/pharmaceutics13071074)
Supplement: Supplementary file 1 [file pharmaceutics-13-01074-s001.zip › pharmaceutics-1221271-supplementary.pdf]

pharmaceutics

Supplementary Materials: Effect of Storage Humidity on Physical Stability of Spray-Dried Naproxen Amorphous Solid Dispersions with Polyvinylpyrrolidone: Two Fluid Nozzle vs. Three Fluid Nozzle

Sonal V. Bhujbal, Yongchao Su, Vaibhav Pathak, Dmitry Y. Zemlyanov, Alex-Anthony Cavallaro, Eric J. Munson, Lynne S. Taylor and Qi (Tony) Zhou

Modulated differential scanning calorimetry (MDSC)

MDSC was also employed for assessing phase immiscibility in samples. The instrument (Q2000 model, TA Instruments, New Castle, DE, USA) was calibrated using indium and tin. About 3–5 mg of sample, placed in a non-hermetically sealed aluminum Tzero pan (TA Instruments, New Castle, DE, USA), was first cooled to 25 °C and then heated to 200 °C at a heating rate of 5 °C/min and modulation of 2 °C every 60 s. The Universal Analysis 2000 software was used to determine the glass transition temperature (Tg) by analyzing reversible heat flow.

However, the results were inconclusive, likely due to the lower drug loading (20% *w*/*w*).

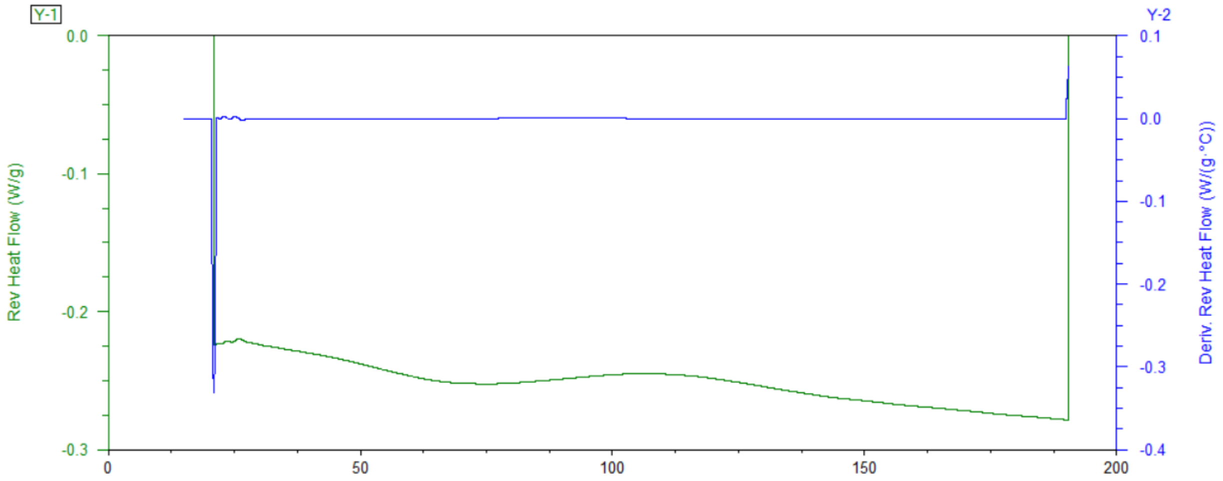

Figure S1. A representative DSC plot of the immiscible (as confirmed by ssNMR) 3FN sample.

Surface area and particle size distribution measurement

The SEM images (Figure. 4) show that the size and morphology of the two powder particles was different. Such differences in size and morphology could affect the surface area of the material. As surface area is known to have an impact on the stability (crystallization) of amorphous material by altering the drug exposure to moisture, particle size and surface area measurements were conducted for the 2FN and 3FN (prepared using two solvents) formulations.

Scanning electron microscopy images were used to determine the particle size of the samples. Three different SEM images were evaluated using the ImageJ software (National

Institute of Health, Rockville, Maryland, USA) and the Martin's diameter of approximately 100 randomly-selected particles was measured for each sample. D_{10}, D_{50} and D_{90} were calculated (Table S1).

Table S1. Particle sizes (*n* = 100) as measured from SEM images using the ImageJ software.

Formulation	Particle size (µm)		
	**D_{10}**	**D_{50}**	**D_{90}**
**2FN**	0.31	0.82	2.43
**3FN**	0.54	1.66	4.92

An ASAP 2020 Plus Physisorption instrument (Micromeritics, Norcross, Georgia, USA) was used to measure Brunauer-Emmett-Teller (BET) surface area of the 2FN and 3FN samples. Each sample was heated at 70 °C under nitrogen gas for 4 h to remove residual moisture and other adsorbed gases. The adsorption of krypton gas on the degassed sample was then measured at p/po = 0.05–0.20 and the resulting BET surface area plot was used to calculate the BET surface area. The BET surface area of the 2FN sample was 0.17 ± 0.01 m^2/g (R2 = 0.9991) and that of the 3FN sample was 0.75 ± 0.01 m^2/g (R2 = 0.9912), respectively. The significantly greater surface area of the 3FN sample as compared to the 2FN sample may have led to greater exposure of the surface drug to the moisture, accelerating its crystallization.
